# Making transparent the accountability deficit in the global climate regime

**DOI:** 10.1038/s44168-025-00264-z

**Published:** 2025-06-26

**Authors:** Aarti Gupta, Max van Deursen

**Affiliations:** 1 Environmental Policy Group, Department of Social Sciences, Wageningen University & Research, Wageningen, The Netherlands; 2 Climate Change Leadership Group, Uppsala University, Uppsala, Sweden

**Keywords:** Climate-change mitigation, Climate change, Politics

## Abstract

Transparency is often extolled as the key means to secure accountability in the global climate regime. In practice, when transparency reports do reveal politically salient information, the use of such information to enhance accountability may not materialize. Whether the advent of the new enhanced transparency framework of the Paris Agreement will change the prospects for accountability-through-transparency in the climate regime remains to be seen.

## Introduction

In the United Nations Framework Convention on Climate Change (UNFCCC) policy context, a widely heard claim is that transparency (in the form of regular reporting and review) is crucial for furthering accountability. The logic is appealing. With the Paris Agreement under the UNFCCC designed around voluntary and nationally determined contributions (NDCs), and with strong compliance and enforcement mechanisms off the table, transparency about such actions is seen as essential to furthering accountability (see Table 1).

**Table 1:** Transparency for accountability: a widely asserted link

*Policy pronouncements*	
European Commission	‘The submission of BTRs is a significant milestone in the implementation of the Paris Agreement, which marks the beginning of a new era of **accountability** […] in the global fight against climate change’^20^
UNFCCC	‘The enhanced transparency framework under the Paris Agreement is an **accountability mechanism**’^21^
United Nations Development Programme	‘Transparency is at the heart of global efforts to fight climate change [by] […] strengthening **accountability**’^22^
Global Environment Facility	‘the ETF will spark mutual trust and **accountability** among nations’^23^
*Academic literature*	
Winkler et al.^24^	‘The transparency framework helps track countries’ progress toward the implementation of their mitigation contributions, which is essential for […] **accountability**’^24^
Rajamani & Bodansky^8^	One of the key characteristics of the Paris Agreement is ‘an emphasis on transparency rather than legal bindingness as the engine to promote […] **accountability**’^8^
Voigt & Gao^25^	‘the provision of mandatory information […] under the transparency framework […] is “the main **mechanism to hold states accountable** for doing what they said they would do”’^25^

Note: emphasis added by the authors.

For example, Bodansky (2016, 311) summarizes clearly the hopes pinned on the transparency-for-accountability link: ‘Since parties’ NDCs are not legally binding, the Paris Agreement’s transparency framework is the main mechanism to hold states accountable for doing what they say. The premise is that peer […] pressure can be as effective as legal obligation in influencing behavior, an issue that has long been debated in the literature’^1^.

Our goal here is to assess if this relationship, long posited but not yet settled, is materializing in practice. Specifically, we ask if UNFCCC transparency obligations, including those pre-dating the Paris Agreement, have helped countries to hold each other accountable for their actions or inactions. This question is hardly ever posed^2,3^, let alone empirically examined, and learning from past experiences is rare^4^.

While the weaknesses of the current climate governance paradigm have been extensively discussed^1,5–8^, the focus is usually on the voluntary nature of targets and the lack of strong enforcement and compliance mechanisms. These deficiencies could be seen to constitute a general lack of accountability in the global climate regime. The role for transparency in securing greater accountability, however, remains more assumed than assessed. Assessing this link is our novel focus.

We understand accountability as a relational concept that has three component elements, all of which need to be present if accountability is to be realized: (1) a specification of who is answerable to whom (that finds agreement from both parties); (2) for what (agreed) standard of performance; and (3) a judgment about whether the standard of performance is being met. If any one of these component elements is missing, it is hard to speak of accountability being furthered^2^.

We argue here that for transparency to generate accountability at least two conditions need to be met: (1) the transparency arrangements should disclose politically salient information (for example, about whether a state or a group of states are adhering to an agreed standard of performance); and (2) there should some engagement with and follow-up on this information (including, ideally, a political judgment about whether a standard is or is not being met) in the multilateral process.

Here we draw on a simple illustrative case to offer novel insights on whether transparency furthers accountability, as understood above. Under the UNFCCC transparency arrangements pre-dating the Paris Agreement, in place through 2024, developed countries were to biennially report on their greenhouse gas emissions, mitigation actions, and finance provided. Subsequently, the UNFCCC Secretariat produced synthesis reports of information provided in these submissions. We analyze the most recent synthesis report published in 2023, as our illustrative case^9^.

We ask two questions: first, does this synthesis report reveal politically salient information? And second, what happens after this information is disclosed? We address these questions in turn below. In concluding, we reflect on what the transition to a new enhanced transparency framework applicable to all countries under the Paris Agreement means for the prospects of furthering accountability through transparency in the global climate regime.

## UNFCCC transparency arrangements: revealing politically salient information

In considering, first, whether transparency arrangements under the UNFCCC generate politically salient information, we address here one compelling instance when they have done so, in the 2023 synthesis report. We should note that we do not aim here to give a balanced summary or comprehensive overview of the synthesis report. Instead, we highlight information contained herein about three controversial and politically salient issues relating to past and future climate actions of developed countries.

First, the 2023 synthesis report reveals, based on self-reported information by developed countries, that emissions of this group of countries decreased by only 17.3% in the three decades between 1990 and 2021 (excluding emissions from Land Use, Land-use Change and Forestry)^9^. It notes that most developed countries achieved their pledged emission reductions for 2020^10^. This information about emission reductions is politically salient, insofar as it relates to who needs to act and by when, in terms of a standard of performance for this group of countries. Although there are few multilaterally agreed parameters for these politically contested aspects in the UNFCCC, the first Global Stocktake does note, for example, that ‘the Intergovernmental Panel on Climate Change had earlier indicated that developed countries must reduce emissions by 25–40% below 1990 levels by 2020’^11^.

Second, and significantly, the synthesis report reveals that emission projections self-reported by developed countries in 2023, based on their policies and measures in place at the time of reporting, showed that no developed country was on track to meet its 2030 NDC target. These self-reported projections also revealed that the collective emissions of developed countries were estimated to increase by 0.5% between 2020 and 2030^9^. Yet it should be noted that the COVID-19 pandemic was responsible for a drop in emissions in 2020. These revelations are politically salient, as they reveal self-reported information about the current and future climate actions of this group of countries in this critical decade. Even if this information can be interpreted in diverse ways, one implication is that developed countries need to formulate and implement additional climate policies before the end of this decade to meet their 2030 targets.

Third, the synthesis report also covers the controversial topic of climate finance, noting that climate finance provision overall has increased, even if the increase has declined in the last reporting years^9^. As a crucial piece of politically salient information, the report noted that self-reported data by developed countries revealed that annual climate finance provided in 2019 and 2020 averaged $51.6 billion^9^. This is politically salient information because it constitutes one of the very few officially quantified amounts revealed by countries themselves in this multilateral process. Moreover, it has a bearing on the self-set goal by developed countries to mobilize (through both public and private sources) a total of $100 billion in climate finance annually by 2020. This self-reported information can be interpreted in diverse ways, but is nonetheless of key relevance for developing country efforts to hold developed countries to account for promised climate finance support.

Taken together, self-reporting by developed countries via the transparency arrangements does reveal politically salient information. This information includes estimated emission reductions by developed countries in the three decades since the adoption of the UNFCCC, projections for emission reductions in this critical decade, and estimates of climate finance provided. This raises a follow-up question: what becomes of this information? Is it taken up and addressed in the political process? Is accountability enhanced as a result?

## Politically salient information disclosed: Does this help to generate accountability?

Here we trace attempts by developing countries to hold developed countries accountable vis-à-vis this politically salient information about climate action and support, and show how these efforts were opposed or deferred within UNFCCC political process. We trace such efforts at account-holding at three UNFCCC climate negotiation sessions following publication of the synthesis report: in Dubai in 2023, Bonn in 2024 and Baku in 2024. At least one of the authors was present in person at each of these sessions described below.

The first opportunity to consider synthesis report findings was at the 28th Conference of the Parties to the UNFCCC (COP28) in Dubai in December 2023. Here, consideration of the report was relegated to a marginal agenda item, and it remained isolated from high-profile issues central to COP28. This included the first Global Stocktake of collective climate performance, which would have been a logical place to engage with its findings. Yet the Global Stocktake outcome merely ‘notes’ gaps in pre-2020 climate actions by developed countries. It stops short of adding quantified information about this gap, even though this information is available in the synthesis report^11^. Even in specific negotiations to ‘consider’ the synthesis report, proposals by developing countries to quantify the gap in past mitigation performance were opposed by developed countries. Given no consensus, the consideration of the synthesis report was postponed to the next negotiation session.

The second opportunity to engage with the synthesis report revelations came at the climate intersessional meeting in Bonn in June 2024. During the opening plenary of this session, Brazil, on behalf of BASIC, a negotiation group including Brazil, South Africa, China, and India, explicitly brought up the synthesis report and expressed concern with its finding that emissions in developed countries are projected to increase between 2020 and 2030^12^. In seeking to hold this group of countries accountable, Brazil sought information on additional measures that developed countries were taking to improve this outlook. Egypt supported Brazil’s intervention and also called for a political deliberation of the findings of the synthesis report on climate finance and its aggregate ‘$51 billion’ figure. Developing countries also pushed, more generally, to adopt text that noted ‘with serious concern’ salient information around mitigation performance and climate finance contained in the synthesis report. Developed countries pushed back on all such efforts, however, noting that the information in the 2023 synthesis report was outdated and did not include their latest policies and measures. Developing countries then proposed a follow-up process in which developed countries would elaborate on additional actions being taken to ensure that NDCs are met. But no agreement could be reached, with the discussion on this report postponed yet again^13^.

As a third opportunity, the matter was taken up at the 29th Conference of the Parties to the UNFCCC (COP29) in Baku in November 2024, but the outcome was similar. Developed countries opposed ‘noting’ any specific information from the synthesis report during political deliberations, arguing that this could constitute cherry picking, and be unbalanced and duplicative. Following an impasse, the issue was deferred again to the 2025 session in Bonn.

These examples suggest that efforts by developing countries to leverage transparency (in the form of self-reporting by developed countries) to hold these countries accountable for their actions and commitments were unsuccessful in this instance. If transparency is widely seen to be the core accountability mechanism in the UNFCCC, this points to an accountability deficit in this contested political arena.

## Conclusion: a continuing accountability deficit in an enhanced transparency world?

Our analysis suggests that the widely asserted link between transparency and accountability does not always materialize in practice. Through the case examined here, we show that pre-Paris UNFCCC transparency arrangements do, in some instances, disclose politically salient information that could be used to generate accountability. Yet attempts to advance accountability based on this disclosed information were deflected and opposed. In effect, ironically, it is through this process of seeking to hold to account on the basis of transparency that the accountability deficit in the global climate regime is itself made transparent.

Where then, does one look for accountability in the global climate regime? Some may argue that accountability is best secured through non-state actor scrutiny, climate litigation or other domestic processes driven by actors and institutions outside of the UNFCCC^14–16^. Within this global regime, however, a tendency is to call for yet more transparency. Here, our analysis above cautions against pinning much hope on this. Yet this is exactly the approach the global climate regime is now taking, with the Paris Agreement’s enhanced transparency framework^17–19^, with ever more detailed reporting applicable to all countries, now becoming operational.

The question then remains: What are the prospects of generating accountability through the new enhanced transparency framework of the Paris Agreement?

We foresee two plausible scenarios.

First, a continuation of an accountability deficit status quo, where some politically salient information may get generated through transparency arrangements but fails to lead to answerability and account-holding within this contested multilateral process.

A second plausible scenario, and one that raises an additional set of important research questions, is that the move to a common transparency framework could change transparency-accountability dynamics. The enhanced transparency framework contains similar reporting requirements applying to all countries, albeit with specific flexibility provisions applying to developing countries who need it in light of their capacities. It is uncertain if, under the Paris Agreement, future synthesis reports by the UNFCCC Secretariat will disaggregate data by developed and developing countries. If not, some of the politically salient information for developed countries as a group highlighted above may not be made officially available. On the other hand, with the light shining on all countries, developed countries might step up efforts to leverage information disclosed by (large) developing counties, to seek to hold them accountable. If so, this raises the question: who gets to leverage politically salient information disclosed through transparency arrangements? Who will be scrutinized by whom, in practice?

We conclude by arguing that empirical research into the widely assumed but empirically largely unestablished relationship between transparency and accountability in the global climate regime continues to be an important area for future research.

## Data Availability

No datasets were generated or analyzed during the current study.
